# Posters Have Limited Utility in Conveying a Message of Antimicrobial Stewardship to Pet Owners

**DOI:** 10.3389/fvets.2019.00421

**Published:** 2019-11-22

**Authors:** Laurel E. Redding, Stephen D. Cole

**Affiliations:** ^1^School of Veterinary Medicine, University of Pennsylvania, Kennett Square, PA, United States; ^2^School of Veterinary Medicine, University of Pennsylvania, Philadelphia, PA, United States

**Keywords:** antimicrobial stewardship, veterinary medicine, education, pet owners, posters

## Abstract

Pet owners frequently administer antimicrobials to their pets and therefore have an important role to play in promoting antimicrobial stewardship in veterinary medicine. However, best methods of educating pet owners about antimicrobial stewardship have yet to be defined. While visual materials such as brochures and posters are often used in health promotion campaigns, their effectiveness in veterinary medicine is unknown. The objective of this study was to determine whether pet owners noticed and retained the message of a poster with an antimicrobial stewardship message placed in veterinary clinic exam rooms. A total of 111 pet owners from five veterinary clinics (three general practices, two low-cost clinics) in the greater Philadelphia area participated in the study. Participants completed a survey asking whether they noticed the poster and if they could paraphrase its message. In a follow-up survey, an antibiotic knowledge score was calculated from answers to questions assessing their knowledge of the poster message. Baseline knowledge was assessed by asking participants to define antibiotic resistance. At the end of the study, veterinarians at participating clinics were interviewed about their experiences with the poster. Only 51 (46.4%) participants noticed the poster, and only 11 (9.9%) could partially or completely reproduce its message. No demographic or clinic-level factors were significantly associated with noticing the poster or recalling its message. Antibiotic knowledge scores were highly correlated (ρ = 0.87, *p* < 0.001) with baseline knowledge and not affected by viewing the poster (*p* = 0.955). Veterinarians expressed skepticism that the poster was effective in conveying a message of judicious antibiotic use to clients and noted no difference in the frequency with which they discussed antibiotic resistance or felt pressured to prescribe antibiotics by their clients. Posters alone will likely have limited impact in conveying a message of judicious antibiotic use to pet owners. However, they might be useful as part of an active, multi-modal education strategy, especially if complemented by veterinarian actions.

## Introduction

Antimicrobials are commonly used in companion animals for therapeutic purposes, and many courses of therapy are given on an outpatient basis by pet owners at home. One component of antimicrobial stewardship in veterinary medicine is education of pet owners about indications for and dosing regimens of antimicrobials (1), with the goal of reducing inappropriate antimicrobial use and promoting acceptance of stewardship (2). While we recently found that pet owners generally have a great deal of trust in their veterinarians, particularly when it comes to prescribing antimicrobials, we also found that a small number of pet owners would challenge a veterinarian who said their pet did not need antibiotics (3).

Similarly, another study found that extrinsic factors related to pet owners, including perceived expectations for antimicrobials, were significant influencers of antimicrobial prescribing among companion animal general practitioners (4). Veterinarians have also reported feeling pressured to prescribe antimicrobials for conditions where their use in unnecessary (4, 5), an occurrence that has also been described in human medicine (6), especially pediatrics (7, 8).

Human patients have been reported to notice and read posters in physicians' waiting rooms (9), but health promotion posters have been shown to be largely ineffective in increasing practices such as requesting influenza vaccination (9) or initiating conversations about weight loss (10). While posters have been part of more extensive campaigns that have achieved some success in decreasing antimicrobial prescribing (11–13), the use of posters alone has been found to have minimal impact: one study found no change in the number of antimicrobial prescriptions for upper respiratory infections in pediatric practices where a poster with an antimicrobial stewardship was placed in the waiting room for a 1-month period (14). Another study noted a decrease in hospitalized patients' self-reported expectations for antibiotics after viewing a poster with an antimicrobial stewardship message (15), but the participants were specifically presented with and directed to read the poster rather than observing it on their own by chance.

To our knowledge, there have been no formal, peer-reviewed studies of the efficacy of posters as a client education tool in a veterinary setting. Several groups have created posters for use in veterinary practice to promote the judicious use of antimicrobials, including the American Veterinary Medical Association [“Careful with Antibiotics, Please” (16)], the British Veterinary Association [“Trust Your Vet” Campaign (17)], and the World Organization for Animal Health [OIE - “Handle Antibiotics with Care” (18)]. The objective of this study was to determine whether pet owners noticed and retained the message of a poster with an antimicrobial stewardship message placed in exam rooms for a 6-month period. A secondary goal was to collect qualitative accounts of veterinarian's experiences with and attitudes toward the poster.

## Materials and Methods

### Recruitment of Pet Owners

A convenience sample of five privately owned small animal veterinary clinics (two low-cost practices and three general practices) was enrolled to participate in the study. A tertiary care referral hospital was approached but declined to participate. A poster with an antimicrobial stewardship message related to upper respiratory infections in dogs and cats produced by the American Veterinary Medical Association was placed in every exam room of the clinics (16). Specifically, the poster stated:

“Cough. Snort. Sniffle. Sneeze. Careful with antibiotics, please. You want your pet to feel better. Antibiotics may or may not be the answer. Antibiotics don't fight viruses, the most common cause of flu-like signs. What will? Good supportive care while your pet's immune system does its job. Find out when antibiotics work-and when they don't. Talk with your veterinarian.”

Veterinarians from the clinics were told that they could discuss the topic with pet owners if they brought it up, but that they did not otherwise need to do anything specific related to the poster. Upon checking out of their appointment (i.e., they no longer had access to the exam room), pet owners were provided with a postcard inviting them to participate in a study with a link and a QR code leading to an online survey. Amazon gift cards ($10 for the first 50 participants, $5 for subsequent participants) were offered as incentives for participating in the study. The posters remained in the clinics for a 6-month period (March-August). The study was approved by the Institutional Review Board of the University of Pennsylvania.

### Surveys

All surveys were administered through Redcap (19). The first online survey led to a picture of the posters with all text blurred out so as to be unreadable and questions relating to the poster and the participant's visit and demographic information (Supplemental Document 1). More specifically, participants were asked whether they noticed the poster. If they indicated that they did, they were asked whether they retained its message and if they could paraphrase it in their own words. The paraphrased answer was assigned a score of 2 points if the message of the poster was reproduced, 1 point if only part of the message was reproduced, and 0 points if none of the message was reproduced.

Once the first survey was completed, participants were invited within 24 h to complete a follow-up online survey assessing knowledge related to antimicrobial resistance, after which they would receive their incentive. The surveys were administered in this sequence to avoid alerting participants to the topic of the poster when asking them to paraphrase its message. This second survey presented the participant with a series of questions related to statements from the poster (“Antibiotics are only needed for treating infections in your pet caused by bacteria,” “Some bacterial infections in dogs and cats get better on their own, without antibiotics,” and “When antibiotics aren't needed, they won't help your pet, and the side effects could cause harm,” Supplemental Document 1). Participants could answer “True,” “False,” or “Don't know” for these statements. A knowledge score was generated from these questions. One point was assigned for each correct true/false statement (i.e., an answer of “True”). To assess pet owners' background knowledge of antimicrobial resistance, the second survey also asked participants to define antibiotic resistance in their own words. A definition score was generated for the following components of the definition: (a) bacteria mentioned as the offending agents (1 point); (b) the drug is no longer effective in treating disease (1 point); (c) resistance is associated with antibiotic use (1 point), for a total possible of 3 points. Scores for the open-ended questions (e.g., paraphrasing of the message of the poster and definition of antibiotic resistance) were assigned by both authors independently. If there was disagreement between the authors, the average of the two scores was assigned. Kappa values were calculated to assess agreement between the two scorers. A Pearson correlation coefficient was calculated to determine the agreement between the knowledge score and the definition score.

### Data Analysis

Descriptive analyses were performed, including calculation of means, medians, standard deviations and ranges, and the distribution of continuous variables was assessed using the Shapiro-Wilk test of normality. Demographic, visit-related factors and knowledge/definition scores were compared among groups (i.e., those who noticed the poster and those who did not) using the chi-squared test, Student's t or Wilcoxon rank sum test. Bivariable analysis was conducted to determine the unadjusted association between (1) demographic/visit factors and visualization of the poster/retention of its message; and (2) demographic/visit factors and the antibiotic knowledge score. Variables that were associated or trending to be associated with the outcome on bivariable analysis (*p* < 0.15), and variables involved in confounding the association between the primary outcome and the factor of interest (i.e., their inclusion in the model resulted in a >15% change in the effect size of the primary association of interest) were added in a stepwise fashion to a mixed effect linear regression model in which visit and demographic factors were fixed effects and the clinic type was a random effect. Model fits were examined using Aikaike Information criteria. All analyses were conducted with Stata 15 (StataCorp, State College TX), with two-sided tests of hypotheses and a *p* < 0.05 as the criterion for statistical significance.

### Veterinary Interviews

Four veterinarians (two practice owners and two associates) and one senior veterinary technician with 9 years of experience who facilitated enrollment of their clinic in the study were interviewed about the experience of having the poster in the clinic at the end of the 6-month period. The following open-ended questions were asked either in person or over the phone.

1. How effective do you think this poster was? Why or why not?
2. Do you ever discuss the issue of antimicrobial resistance with your clients? Did you notice an increase in how much you talk about it when the poster was up?
3. Do you ever feel pressured by your clients to prescribe antibiotics? Did this change when the poster was up?
4. Did anyone bring up the poster with you? What was that interaction like?
5. What other tools do you think would be effective in conveying a message of judicious antibiotic use to clients?

The veterinary technician was asked to respond in reference to both herself and what she observed in her interactions with veterinarians in her clinic. Responses were transcribed and analyzed using conventional content analysis (20).

## Results

### Survey Respondents

A total of 111 people participated in the study, including 52 from the low-cost clinics and 59 from the general practices. Most of the participants were from only two clinics (general practice 1, *n* = 27, 24.3%; and low-cost clinic 1, *n* = 12, 10.8%), with the three remaining clinics contributing very few participants (*n* = 14, 12.6%). The remaining 58 participants (*n* = 52%) did not indicate which clinic they attended. The clinics that contributed the most participants had either one or no other posters in their waiting rooms. Sixty-three participants (67.7%) were female. More information on the participants is presented in Table 1.

**Table 1:** Demographic and visit-level characteristics of pet owners who participated in a study about a poster with an antimicrobial stewardship message at five veterinary clinics in the greater Philadelphia area.

	**All participants (*n* = 111)**	**Participants who did not notice the poster (*n* = 60)**	**Participants who noticed the poster (*n* = 51)**	***P*-value**
**Sex (*****n*****, %)**				
Male	30 (27.0)	13 (21.7)	17 (33.3)	0.121
Female	63 (56.8)	37 (61.7)	26 (51.0)	
No response	18 (16.2)	10 (16.7)	8 (15.7)	
Mean (SD) participant age (years)	42.8 (16.2)	41.0 (16.0)	44.1 (15.9)	0.314
**Education**				
- Completed high school	26 (23.9)	15 (25.0)	11 (21.6)	0.670
- Some college but no degree	20 (18.4)	10 (16.7)	10 (19.6)	
- Associate or bachelors degree	43 (39.3)	23 (38.3)	20 (39.2)	
- Graduate or professional degree	20 (18.4)	11 (18.3)	9 (17.7)	
- No response	2 (1.80)	1 (1.7)	1 (2.0)	
Median (IQR) time spent in the exam room in minutes	20 (12–30)	15 (10–30)	20 (15–30)	0.332
Number of pets [median (IQR)]	2 (1–3)	2 (1–3)	2 (1–3)	0.629
Mean (SD) antibiotic knowledge score (points, out of 6 possible)	3.4 (1.7)	3.3 (1.9)	3.4 (1.6)	0.850

Fifty-one (46.4%) participants reported having noticed the poster, and this proportion was similar in the low-cost (26/52, 50.0%) and general practice (25/59, 42.4%) clinics (*p* = 0.421). Only one person (2.0%) who noticed the poster stated that they discussed the topic of the poster with their veterinarian. Of the 51 participants who noticed the poster, 13 (25.5%) stated that it was not the first time they had seen the poster. No demographic or visit-level factors were statistically significantly associated with noticing the poster (Table 1). Most (32/51, 62.8%) of the people who noticed the poster said they did not remember what the message of the poster was, while 6 (11.8%) said they did remember and 13 said they “sort of” remembered the message of the poster (Table 2). Of these 19 people, 8 (42.1%) were able to paraphrase part of the message of the poster (i.e., score of 1/2), while only 3 (15.8%) could reproduce the message of the poster in its entirety (i.e., score of 2/2). There was substantial interrater agreement (21) for the scores evaluating whether the message of the poster was retained (κ = 0.78, *p* < 0.001). Paraphrasing of the poster message for each respondent and their associated score is presented in Table 2. Of the 13 participants who said they had seen the poster before, 10 (76.9%) stated that they did not remember the message of the poster, while the remaining 3 (23.1%) said they “sort of” remembered it. There was no significant difference in scores between participants who reported having seen the poster before (median score 0 points) and those who reported never having seen the poster before (median score 1) (*p* = 0.390).

**Table 2:** Paraphrasing of the content of a poster with an antimicrobial stewardship message by pet owners who noticed the poster in the exam room of one of five veterinary clinics in the greater Philadelphia area.

**Participant's stated retention of the poster's message**	**Paraphrased poster message**	**Poster retention score out of 2 points**
Participants who claimed to have remembered the message of the poster (*n* = 6)	“It basically told you to be careful giving antibiotics to your pets. I actually saw the one with the dog and it was advising you to use precautions with antibiotics”	2
	“Overuse of antibiotics”	2
	“Be careful with giving antibiotics for cold and flu like symptoms as the viruses can become immune. Speak with the doctor about the best course of action for your pet”	2
	“Pet antibiotics”	1
	“Information about antibiotics”	1
	“It's tick season. Ticks are hunting to attack your pet”	0
Participants who claimed to have “sort of” remembered the message of the poster (*n* = 13)	“Antibiotics are not for every issue”	2
	“Be careful with antibiotics”	2
	“Antibiotics?”	1
	“Coughing, sneezing and antibiotics”	1
	“Careful how you treat your pet. May not need treatment”	1
	“Pet meds”	0
	“Sneezing”	0
	“Please adopt me”	0
	“Something about rabies vaccines”	0
	“Help support animal welfare and donate.”	0
	“Micro-chipping”	0
	“Healthy pets are a joy to you”	0

### Antibiotic Knowledge Scores

Of the 111 participants who completed the first survey, 91 (82.0%) filled out the second follow-up survey. All 91 participants answered all of the True/False questions, but only 83 participants provided a definition of antibiotic resistance. There was substantial interrater agreement (21) for the scores associated with the definition of antibiotic resistance (κ = 0.65, *p* < 0.001). Most people answered the true/false questions correctly (Table 3), and there was no significant difference in knowledge score between participants who noticed and did not notice the poster (1.96 vs. 1.95, *p* = 0.955). The mean (SD) score for the definition of antimicrobial resistance was 1.38 (1.03) out of 3 possible points, and this value was similar for participants who noticed and did not notice the poster (*p* = 0.693, Table 3). The antibiotic knowledge score was significantly associated with the definition score (ρ = 0.87, *p* < 0.001).

**Table 3:** Responses to questions on antibiotics and antibiotic resistance posed to pet owners who sat in a clinic exam room with a poster with an antimicrobial stewardship message in one of five clinics in the greater Philadelphia area.

	**Number (proportion of respondents)**			
**Question and point value**	**All respondents (*n* = 91)^**a**^**	**Participants who did not notice the poster (*n* = 51)**	**Participants who noticed the poster (*n* = 40)**	***P*-value**
Antibiotics are only needed for treating infections in your pet caused by bacteria (1 pt)				
- True	67 (73.6)	38 (74.5)	29 (72.5)	0.537
- False	17 (18.7)	8 (15.7)	9 (22.5)	
- Don't know	7 (7.7)	5 (9.8)	2 (5.0)	
Some bacterial infections in dogs and cats get better on their own, without antibiotics (1 pt)				
- True	48 (52.8)	27 (52.9)	21 (52.5)	0.375
- False	22 (24.2)	10 (19.6)	12 (30.0)	
- Don't know	21 (23.1)	14 (27.5)	7 (17.5)	
When antibiotics aren't needed, they won't help your pet, and the side effects could cause harm (1 pt)				
- True	62 (68.9)	35 (68.6)	27 (69.2)	0.998
- False	7 (7.8)	4 (7.8)	3 (7.7)	
- Don't know	21 (23.3)	12 (23.5)	9 (23.1)	
Cumulative knowledge score				
- 0 points	8 (8.9)	5 (9.8)	3 (7.7)	0.388
- 1 point	23 (25.6)	14 (27.5)	9 (23.1)	
- 2 point	24 (26.7)	10 (19.6)	14 (35.9)	
- 3 points	35 (38.9)	22 (43.1)	13 (33.3)	
Mean (SD) knowledge score	1.96 (1.00)	1.96 (1.06)	1.95 (0.94)	0.955
Mean (SD) antibiotic resistance definition score (out of 2 points)	1.38 (1.0)	1.35 (1.1)	1.42 (0.94)	0.693

a *This number represents the participants who completed both the first and second survey and is therefore smaller than the total number of study participants (n = 111)*.

On univariable analysis, education and prior viewing of the poster were significantly associated with the knowledge score (Table 4): for each increase in level of education, the knowledge score increased by 0.17 points (*p* = 0.017), and having seen the poster was associated with a decrease in knowledge score of 0.72 points (*p* = 0.040). On multivariable analysis, when adjusting for the definition score, neither education nor prior viewing of the poster were significantly associated with the knowledge score (Table 4).

**Table 4:** Association between antibiotic resistance knowledge score and participant demographic/visit factors of pet owners in the greater Philadelphia area who sat in a clinic exam room with a poster with an antimicrobial stewardship message.

**Factor**	**Univariable analysis (*****n*** **=** **111)**			**Multivariable analysis (*****n*** **=** **83)**^****a****^		
	**Coefficient**	**95% CI**	***P*-value**	**Coefficient**	**95% CI**	***P*-value**
Education	0.17	0.30–0.31	0.017	0.14	−0.06–0.35	0.167
Saw poster previously	−0.72	−1.41–(−0.03)	0.040	−0.57	−1.22–0.83	0.117
Antibiotic resistance definition score	0.51	0.33–0.67	<0.001	0.23	−0.10–0.56	0.168

a *This number represents the participants who fully completed both the first and second survey (including providing a definition for antibiotic resistance) and is therefore smaller than the total number of study participants (n = 111)*.

### Veterinarian Responses

All of the veterinarians and the senior veterinary technician stated that they did not find the poster to be effective in conveying a message of antimicrobial stewardship because they suspected few people noticed it. Reasons for this varied. One general practice veterinarian stated:

“I think most people just don't look at posters now. Probably because people have phones, right? When they're in [an exam room] by them self, they don't look around, they just look at their phone.”

Another general practice veterinarian said:

“Our clients are not in the room for very long, […] and if they are, it's because their animal is usually pretty sick, and then they're just not paying attention to any of that.”

The veterinary technician stated:

“Our rooms tend to be poster-heavy [5-6 posters per room], so it may have blended in with the others.”

One of the veterinarians mentioned that the poster was helpful for herself in reminding her to discuss the topic of antimicrobial resistance with her clients when the situation came up.

Only two general-practice veterinarians stated that any clients discussed the poster with them. One veterinarian stated that a few people did:

“They [the clients] would say something like ‘Oh yeah, this is interesting! We've heard about this in people, you know, the overuse of antibiotics,’ […] For them it was more of a curiosity learning experience.”

The other veterinarian said that one client pointed the poster out but no discussion of its topic ensued.

All of the veterinary personnel mentioned that they did occasionally discuss the topic of antimicrobial resistance with their clients—mostly in the context of chronic skin, ear, or urinary tract infections, or to explain why a different choice of antimicrobial is needed, or to underscore why the entire course of the drug regimen should be completed. None of the veterinary personnel noticed a change in the frequency with which they discussed this topic while the poster was up.

All of the veterinarians mentioned that they sometimes or often felt pressured to prescribe antibiotics by their clients. One low-cost clinic veterinarian stated:

“[I] constantly [feel pressure to prescribe antibiotics] for clearly viral infections and abnormal urinary signs, especially in cases where clients have limited financial means. […] I had one person demand I give her cat Baytril last week because she was told it was the best antibiotic ever.”

One general practice veterinarian said:

“Oh, all the time! I think everyone does [feel pressured to prescribe antibiotics], and they'd be lying if they told you otherwise. […] I think a lot of the people [pet owners] just want to do something [for their sick pet], […] but if they were somewhere else where the other doctor always did this [prescribe an antibiotic] and it worked, that's where it particularly becomes an issue.”

None of the veterinary personnel noted a difference in the frequency with which this type of pressure occurred while the poster was up.

When asked what other tools would be effective in conveying a message of judicious antibiotic use to clients, three of the veterinarians and the veterinary technician thought that a brochure would be better than a poster, though they all expressed doubts that brochures they distributed to their clients were actually read. One of the general practice veterinarians stated that she frequently received interest from clients for a poster about poisonous plants and thought that clients paid more attention to posters with lists. She also stated:

“I think people like stories. More of ‘this happened because of that’, or ‘We could have avoided this if we didn't use that’. More of ‘Look at Fluffy, Fluffy had overexposure to antibiotics and this is what resulted because now we have this infection that we can't treat’. I think that that hits home more. […] People remember [stories].”

## Discussion

In this study, we found that a poster placed in the exam rooms of veterinary clinics was not very effective in conveying a message of antimicrobial stewardship. Fewer than half of the participants (46.9%) noticed the poster, and even fewer (10/111, 9%) retained its message. Knowledge of antibiotics was not increased by viewing of the poster, and seeing the poster more than once actually resulted in a decreased antibiotic knowledge score.

The ultimate goal of educational visual material such as posters is to inform or change behavior. The stated goal of the AVMA posters was to “help explain why your clients' animals don't need antibiotics for most ailments” (16). A presumed desired outcome of this poster and other posters with a similar message is to decrease the incidence of clients requesting antibiotics or increase pet owners' acceptance of a veterinarian's decision not to prescribe antibiotics when they are not necessary. The assumptions underlying such objectives are that clients do in fact pressure veterinarians to prescribe antibiotics, that they will see a poster and retain its message, and that this newly acquired information will change their behavior. However, it is very difficult to measure such an outcome. We did not have access to information on the number of antimicrobial prescriptions in participating clinics and we could not assess pet owners' propensity to request antibiotics. We therefore measured the actions that would occur “upstream” of a poster-induced change in behavior with regards to antibiotic prescribing—actual viewing of the poster and retention of its message. We also sought to determine whether people who noticed the poster were more likely to be able to correctly answer questions relating to its content. While we could not assess pet owners' baseline knowledge of or expectations for antibiotics prior to the first survey without alerting them to the message of the poster, we did use a proxy measure of baseline knowledge by asking them to define antibiotic resistance in their own words. Finally, we assessed whether veterinarians perceived a change in how frequently they discussed the topic of antimicrobial resistance with clients or felt pressured to prescribe antibiotics while the poster was up.

It remains unclear why viewing of the poster was not associated with an increased antibiotic knowledge score and why multiple viewings of the poster were negatively associated with the knowledge score. It could be that, given the relatively high number of people who answered the True/False questions correctly, we were underpowered to detect a difference in score for people who did or did not notice the poster. The significant association between antibiotic knowledge and education on univariable analysis but not on multivariable analysis is likely due to the fact that we were underpowered to detect the true adjusted effect: because only 83 participants completed both surveys and provided a definition of antibiotic resistance, the sample size for the multivariable analysis was smaller than the sample size for the univariable analysis.

It is reasonable to conclude that if the poster was ineffective in increasing knowledge of pet owners with regards to judicious use of antibiotics, it is unlikely that the poster could change behavior. Moreover, it is still questionable how widespread the undesirable behavior that the poster is presumably intended to address (pressuring veterinarians for antibiotics) is. In contrast to what was anecdotally reported by veterinarians in this study (i.e., that they are often pressured to prescribe antibiotics), other studies suggest that pressuring of veterinarians for antibiotics by pet owners occurs infrequently (3, 5) and that there is a dissonance between perceptions of veterinarians and pet owners with regard to expectations for antibiotics (5), similar to that documented in pediatrics (8, 22). Nevertheless, a poster could be useful in providing an inexpensive and simple vehicle for the passive absorption of a message of judicious antibiotic use, even if it only reaches 9% of pet owners. Moreover, one of the veterinarians reported finding the poster useful as a reminder to discuss the issue of judicious antibiotic use more frequently with her clients. Such visual material could therefore be useful under certain circumstances. However, because antimicrobial resistance tends to be a nebulous and future threat that is often discounted in the face of more immediate concerns (23), pet owners may not pay heed to such a message when concerned about their pets' health, and veterinarians might be reticent to cede valuable exam room wall “real estate” to such a poster. The skepticism expressed by all of the veterinary personnel in this study that the poster was effective underscores this possibility. Future studies are needed to investigate the best practices for veterinarians to initiate conversations with clients about antimicrobial resistance.

The lack of an association between viewing of the poster and improved antibiotic knowledge score is consistent with findings in human medicine that antimicrobial stewardship interventions involving patient or prescriber education alone are limited in their effectiveness (11–13, 24). In fact, the guidelines of the Infectious Disease Society of America for implementing antimicrobial stewardship programs in human settings include a recommendation against solely relying on didactic, passive education as an approach to improving antimicrobial use (25). The recommendations suggest that education is best combined with other approaches such as prospective audit and feedback of providers. Several studies have shown that multimodal approaches to patient/pet owner and clinician education can decrease antimicrobial prescribing (26–28) and improve patient (29) and pet owner (30) satisfaction. However, additional studies are needed to investigate the effectiveness of such multimodal approaches in the specific context of antimicrobial stewardship in veterinary medicine.

There were some limitations to the study. First, only a small number of participants from each clinic enrolled, despite the monetary incentive for participation, the simplicity of participation, and the lengthy time period during which the poster was in place. We do not know what proportion of pet owners visiting the clinics our numbers represent, as the participating clinics were either unable or unwilling to provide us with the total number of pet owners who visited their clinics during the study. We therefore do not know how representative the sample of study participants was. However, it is likely that the small sample size limits the generalizability of the results of the study, might have introduced selection bias, and might not accurately reflect the true number of people who saw the poster and retained its message. Second, we do not know the reasons why pet owners were at the clinic (e.g., sick visits vs. preventive care) or whether they were prescribed antibiotics during their visit, and pet owners may have been more or less likely to view the poster and retain its message based on these factors. Third, the use of true/false questions to assess a participant's knowledge of antibiotics after viewing the poster could result in artificially high scores if people guessed the answers correctly. Fourth, a participant's definition of antibiotic resistance might not be an accurate reflection of their “background” knowledge, especially if a person looked up the definition prior to answering the question. Fifth, the posters were up during the spring and summer (March-August), whereas respiratory disease in dogs and cats tends to occur in the fall and winter (31, 32). Owners may therefore not have perceived the message of the poster as relevant and might have been less likely to remember it. Finally, we did not know how long pet owners waited to complete the survey after their visit. If the interval between the visit and completion of the survey was large, recall of the poster's message could have been affected. Future studies are needed to investigate other methods of presenting antimicrobial stewardship material to pet owners and to evaluate whether they can effect change in behavior.

## Conclusions

It is likely that, as has been found in human medicine (14, 33), posters alone will be ineffective in conveying a message of judicious antibiotic use in veterinary medicine. We suggest that posters might have some utility if they are a part of an active, multi-modal education campaign that could also involve, as suggested by the veterinarians in this study, brochures, educational stories, and video material (12, 34, 35). Such a strategy is even more likely to be successful if it is coupled with stewardship actions conducted by the veterinarian (35).

## Data Availability Statement

The datasets generated for this study are available on request to the corresponding author.

## Ethics Statement

The studies involving human participants were reviewed and approved by University of Pennsylvania Institutional Review Board. Written informed consent for participation was not required for this study in accordance with the national legislation and the institutional requirements.

## Author Contributions

LR and SC contributed to the design of the study and writing of the manuscript. LR conducted all analyses.

### Conflict of Interest

The authors declare that the research was conducted in the absence of any commercial or financial relationships that could be construed as a potential conflict of interest.
