# Supplementary material for: Posters Have Limited Utility in Conveying a Message of Antimicrobial Stewardship to Pet Owners
Source: Front Vet Sci. 2019 Nov 22;6:421. doi: 10.3389/fvets.2019.00421 (PMC6883349; doi:10.3389/fvets.2019.00421)
Supplement: Supplementary file 1 [file Table_1.DOCX]

SURVEY 1


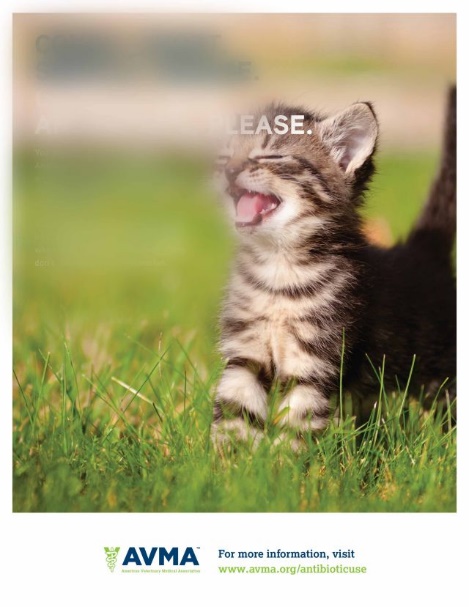

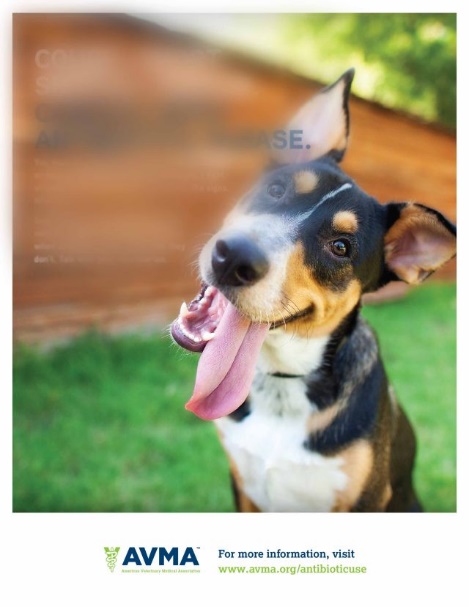


Did you notice this poster when you were in the examination room?

Yes No

Was this the first time you noticed the poster?

Yes No

Do you remember what the message of the poster was

Yes No Sort of

What, in your own words, was the message of the poster?

_____________________________________________________________________

Did you discuss the topic of the poster with your veterinarian?

Yes No

Approximately how many minutes were you in the waiting room?

__________________

What is your age?

__________________

Are you -

Male Female

What type of pets do you have (check all that apply)

Dogs Cats Other________________

How many pets do you have?

__________________

What is your highest level of education completed?

Less than high school

High school or equivalent (GED)

Some college, but no degree

Associate or bachelor’s degree

Graduate or professional degree

Please enter your email address if you would like to be eligible to receive a gift card.

__________________________

SURVEY 2

Thank you for participating in our study! Please answer the following questions. Upon completion, you will receive your gift card within 3 business days.

**Please read the following statements and respond whether you think they are true or false. If you are not sure, please indicate “Don’t know”.**

**Antibiotics are only needed for treating infections in your pet caused by bacteria**

**True False Don’t know**

**Some bacterial infections in dogs and cats get better on their own, without antibiotics.**

**True False Don’t know**

**When antibiotics aren’t needed, they won’t help your pet, and the side effects could still cause harm.**

**True False Don’t know**

What, in your own words, is antimicrobial resistance?

__________________________________
